# Real-Time PCR Detection of *Alicyclobacillus acidoterrestris* in Fruit Juice: Method Validation and Implications for Guaiacol-Related Spoilage

**DOI:** 10.3390/foods15101672

**Published:** 2026-05-11

**Authors:** Shih-Ling Lin, Melanie M. Valdrez, Shun-Hsien Chang

**Affiliations:** 1 Institute of Food Safety and Risk Management, National Taiwan Ocean University, Keelung 20224, Taiwan; 2 Food and Drug Administration, Ministry of Health and Welfare, Taipei City 115021, Taiwan; 3 Marine Biotechnology and Environmental Ecology Sustainability, National Taiwan Ocean University, Keelung 20224, Taiwan; 4 Center for Marine Bioscience and Biotechnology, National Taiwan Ocean University, Keelung 20224, Taiwan

**Keywords:** *Alicyclobacillus acidoterrestris*, real-time PCR, *vdcC* gene, fruit juice, guaiacol

## Abstract

In this study, a SYBR Green-based real-time PCR workflow targeting the *vdcC* gene was optimized and validated for rapid detection of *Alicyclobacillus acidoterrestris* in fruit juice. A commercial DNA extraction kit showed the best performance, achieving a limit of detection (LOD) of 2 Log CFU/mL, whereas microwave-based extraction (30 s) provided a rapid alternative with an LOD of 3 Log CFU/mL. The *vdcC* primer set enabled clear discrimination of *A. acidoterrestris* from closely related species based on melting curve analysis. The method was successfully applied to orange, apple, and grape juice matrices, as well as to 30 commercial juice samples. Guaiacol analysis further indicated that the presence of *A. acidoterrestris* DNA did not necessarily correspond to active spoilage. Overall, this study provides a systematically optimized and practically validated workflow for monitoring *A. acidoterrestris* in fruit juice systems.

## 1. Introduction

*Alicyclobacillus acidoterrestris* is a thermo-acidophilic, spore-forming bacterium widely recognized as a major spoilage microorganism in fruit juice products. Unlike many foodborne bacteria, *A. acidoterrestris* does not pose a direct health risk; however, it can survive pasteurization and subsequently germinate during storage, leading to the production of off-flavor compounds such as guaiacol [1]. This compound imparts a medicinal or smoky odor, resulting in significant quality deterioration and economic losses in the juice industry. Due to its acid tolerance and heat-resistant spores, controlling *A. acidoterrestris* remains a persistent challenge in juice processing and storage systems [2].

Conventional detection methods for *A. acidoterrestris*, including culture-based techniques, are time-consuming and may fail to detect sublethal injured or non-culturable cells. In recent years, molecular methods such as real-time polymerase chain reaction (real-time PCR) have been increasingly applied for rapid and sensitive detection of foodborne microorganisms [3]. Among these, SYBR Green-based real-time PCR offers advantages in simplicity and cost-effectiveness; however, its non-specific binding to double-stranded DNA may lead to false-positive signals, necessitating additional validation through melting curve analysis. Furthermore, the presence of complex food matrices, such as fruit juices, may introduce PCR inhibitors that affect detection sensitivity and accuracy, highlighting the importance of optimized DNA extraction and assay conditions [4]. Compared with conventional PCR, real-time PCR provides improved sensitivity, shorter time-to-result, and semi-quantitative information through Ct values. In addition, although droplet digital PCR may offer higher analytical precision, real-time PCR remains more accessible and practical for routine screening in food microbiology laboratories and industrial quality control.

This study does not claim to introduce a new molecular target or to be the first application of real-time PCR to *A. acidoterrestris* in fruit juice. Instead, its contribution lies in the development of a systematically optimized workflow that integrates (i) a comparative evaluation of five DNA extraction approaches, including rapid microwave lysis, (ii) a SYBR Green-based *vdcC* assay capable of species-level discrimination through melting-curve analysis, and (iii) validation in three juice matrices and commercial juice samples with parallel guaiacol determination. To our knowledge, this combination has not been collectively addressed in previous studies.

## 2. Materials and Methods

### 2.1. Bacterial Strains and Culture Conditions

A total of 31 bacterial strains were used in this study. Four *Alicyclobacillus* reference strains, including *Alicyclobacillus acidoterrestris* BCRC 17660, *A. cycloheptanicus* BCRC 17522, *A. hesperidum* BCRC 17524, and *A. acidocaldarius* BCRC 14685, were obtained from the Food Industry Research and Development Institute (Hsinchu, Taiwan). In addition, 27 non-target strains were provided by the Taiwan Food and Drug Administration (TFDA, Taipei, Taiwan), including *Bacillus cereus* (BCRC 10876, BCRC 10446, BCRC 10603), *Bacillus coagulans* (BCRC 14652), *Bacillus licheniformis* (M64-180800076, M64-180800251), *Bacillus* spp. (M64-180800074), *Bacillus pumilus* (M64-180800075), *Bacillus subtilis* (BCRC 10447, BCRC 15411), *Bacillus thuringiensis* (BCRC 14683, BCRC 15660), *Bacillus vallismortis* (M64-180600142, M64-180800074), *Escherichia coli* (BCRC 10675, BCRC 11509, BCRC 11634, BCRC 13084, BCRC 13091, BCRC 15375), *Listeria monocytogenes* (BCRC 14845, BCRC 14847), *Salmonella typhimurium* (BCRC 10312, BCRC 10747, BCRC 12947), and *Staphylococcus aureus* (BCRC 12653, BCRC 12654). For activation, each *Alicyclobacillus* strain was inoculated into 5 mL of BAT broth and incubated at 50 °C for 24 h. This activation procedure was repeated twice to ensure optimal physiological activity. For culture preparation, 10 μL of the twice-activated culture was inoculated into BAT broth (pH 4.0) and incubated at 50 °C with shaking at 120 rpm for 24 h to achieve a cell concentration of approximately 8 Log CFU/mL or higher. Viable counts were determined by spreading 0.1 mL of culture onto BAT agar plates, followed by incubation at 50 °C for 24 h.

### 2.2. DNA Extraction Methods

#### 2.2.1. Heat-Based DNA Extraction

Heat-based DNA extraction was carried out by resuspending the pellet in sterile deionized water, followed by treatment using a heating shaker (70–99 °C, 10–30 min) [5], water bath (70–99 °C, 10–30 min) [6], or microwave irradiation (800 W, 10–60 s) [6]. After treatment, samples were centrifuged and the supernatant was collected as crude DNA for further analysis.

#### 2.2.2. Ethanol Precipitation Method

DNA extraction by ethanol precipitation was performed using a modified ethanol precipitation protocol [7]. Briefly, 1 mL of bacterial culture was centrifuged at 5000× *g* for 10 min, and the supernatant was discarded. The pellet was resuspended in 180 μL of enzymatic lysis buffer and incubated at 37 °C for 30 min to facilitate cell lysis. Subsequently, 1 mL of absolute ethanol was added, and the mixture was vortexed thoroughly. The samples were then incubated at −70 °C for 30 min to enhance DNA precipitation. After incubation, samples were centrifuged at 12,000× *g* for 20 min at 4 °C, and the supernatant was discarded. The DNA pellet was air-dried at room temperature for 10 min and resuspended in 100 μL of Buffer AE to obtain the final DNA solution. The extracted DNA was stored in microcentrifuge tubes until subsequent analysis.

#### 2.2.3. Commercial DNA Extraction Kit

Genomic DNA was extracted using the DNeasy Blood & Tissue Kit (Qiagen, Hilden, Germany) according to the manufacturer’s instructions with minor modifications. Briefly, 1 mL of enrichment culture was transferred into a 1.5 mL microcentrifuge tube and centrifuged at 5000× *g* for 10 min. The supernatant was discarded, and the pellet was resuspended in 180 μL of enzymatic lysis buffer, followed by incubation at 37 °C for 30 min. Subsequently, 25 μL of proteinase K and 200 μL of Buffer AL were added, mixed thoroughly, and incubated at 56 °C for 30 min. Then, 200 μL of ethanol was added and the mixture was mixed well. The mixture was transferred to a DNeasy Mini spin column placed in a 2 mL collection tube and centrifuged at 7100× *g* for 1 min.

The flow-through was discarded, and the column was transferred to a new collection tube. The column was washed with 500 μL Buffer AW1 and centrifuged at 7100× *g* for 1 min, followed by a second wash with 500 μL Buffer AW2 and centrifugation at 14,000 rpm for 3 min. After removing the flow-through, the column was placed into a clean microcentrifuge tube. DNA was eluted by adding 200 μL Buffer AE, incubating at room temperature for 1 min, and centrifuging at 7100× *g* for 1 min. The eluted DNA was collected and stored for subsequent analysis.

#### 2.2.4. Determination of DNA Concentration and Purity

DNA concentration and purity were determined according to Koetsier and Cantor [8] with minor modifications. Briefly, 1 μL of DNA solution was analyzed using a microvolume spectrophotometer to measure absorbance at 260 nm and 280 nm. DNA concentration was calculated based on the absorbance at 260 nm using the following equation:

DNA concentration (ng/μL) = A_260_ × dilution factor × 50.

The A_260_/A_280_ ratio was used to evaluate DNA purity. A ratio between 1.7 and 2.0 was considered indicative of acceptable purity.

### 2.3. Optimization of Real-Time PCR Conditions

#### 2.3.1. Primer Sets

Primer sets targeting different genetic markers were selected based on Wang et al. [9] with minor modifications. Three primer sets, including *vdcC*, A1-92-3, and *NAtb*, were synthesized and evaluated. Four *Alicyclobacillus* strains—*Alicyclobacillus acidoterrestris* BCRC 17660, *A. cycloheptanicus* BCRC 17522, *A. hesperidum* BCRC 17524, and *A. acidocaldarius* BCRC 14685—were used for real-time PCR analysis.

#### 2.3.2. PCR Reagents

Two commercial qPCR master mixes were evaluated, including TB Green Premix Ex Taq II (Takara Bio Inc., Kusatsu, Japan) and KAPA SYBR FAST qPCR Master Mix (Kapa Biosystems, Wilmington, MA, USA). The reaction conditions for each reagent were established according to the manufacturers’ instructions.

#### 2.3.3. Specificity Test

Specificity testing was conducted following the method of Wang et al. [10] with minor modifications. Four *Alicyclobacillus* strains were cultured in BAT broth at 50 °C with shaking at 120 rpm for 24 h. In addition, 27 non-target bacterial strains were cultured on TSA at 35 °C for 24 h. DNA was extracted using both the DNeasy Blood & Tissue Kit and the microwave method (30 s treatment). The extracted DNA was subjected to real-time PCR analysis to evaluate assay specificity.

#### 2.3.4. Limit of Detection (LOD)

The limit of detection was determined based on Wang et al. [10] with minor modifications. Briefly, 10 μL of purified *A. acidoterrestris* BCRC 17660 culture was inoculated into BAT broth (pH 4.0) and incubated at 50 °C with shaking at 120 rpm for 24 h to obtain the initial culture. The culture was serially diluted (10-fold) up to 10^−8^. Bacterial counts were determined using the plate count method. DNA was extracted using both the DNeasy kit and microwave treatment (30 s), followed by real-time PCR analysis to determine the detection limit.

### 2.4. Juice Matrix Spiking Test

The matrix effect was evaluated according to Wang et al. [9] with minor modifications. Briefly, *A. acidoterrestris* BCRC 17660 was cultured as described above to obtain an initial concentration of approximately 8 Log CFU/mL. The culture was serially diluted (10-fold), and 2 mL of each dilution was added to 18 mL of different juice matrices, including orange juice, apple juice, and grape juice. The mixtures were homogenized thoroughly. From each sample, 1 mL was collected for DNA extraction using both the DNeasy kit and microwave treatment (30 s), followed by real-time PCR analysis.

### 2.5. Market Survey

A total of 30 juice samples were collected from supermarkets, traditional markets, beverage shops, and fresh orange juice vending machines in Taipei, Taiwan. The samples included reconstituted juice, commercially sterilized juice, and freshly prepared juice. For each sample, 1 mL of juice and enrichment culture (after 0 and 1 day of incubation) were subjected to real-time PCR analysis. In addition, guaiacol content was determined using HPLC. Samples were analyzed at day 0 and after 1 day of incubation to distinguish between the presence of bacterial DNA and the potential for bacterial growth, thereby allowing for differentiation between viable and non-viable cells.

### 2.6. Conventional Culture Method

For conventional microbiological analysis, 1 mL of juice sample was added to 9 mL of BAT broth and incubated at 50 °C for 24 h. After enrichment, 0.1 mL of the culture was spread onto BAT agar plates using a sterile spreader. The plates were incubated in an inverted position at 50 °C for 24 h, and the colonies were subsequently counted to determine the bacterial population.

### 2.7. DNA Sequencing and Sequence Alignment

Juice samples that showed positive amplification in real-time PCR analysis were subjected to sequencing for confirmation. The PCR products were purified and sequenced. The obtained sequences were uploaded to the NCBI BLAST 2.17.0. platform and compared against the GenBank database for sequence alignment. Species identification was confirmed based on sequence similarity to *Alicyclobacillus acidoterrestris* reference sequences.

### 2.8. Determination of Guaiacol Content by HPLC

Guaiacol content was determined by HPLC as described previously [11], with minor modifications. Briefly, 2 mL of juice sample or enrichment culture was subjected to pre-treatment by centrifugation at 10,000 rpm for 10 min. The supernatant was collected, diluted 10-fold, and filtered through a 0.22 μm membrane filter prior to analysis. HPLC analysis was performed using a C18 column (5 μm, 4.6 mm × 150 mm). The column temperature was maintained at 40 °C, and detection was carried out at 280 nm. The injection volume was 10 μL, and the flow rate was set at 1 mL/min. The mobile phase consisted of solvent A (deionized water containing 0.1% formic acid) and solvent B (100% methanol). Separation was achieved using a gradient elution program as follows: the initial composition was 60% A and 40% B, which was maintained for 2 min; the composition was then linearly changed to 50% A and 50% B at 10 min; subsequently, it was rapidly adjusted to 10% A and 90% B at 10.5 min and held until 13.5 min; finally, the system was returned to the initial condition (60% A and 40% B) at 14 min and equilibrated until 17 min.

### 2.9. Statistical Analysis

Statistical analysis was performed using SPSS version 20 (IBM Corp., Armonk, NY, USA). All experimental data were expressed as mean ± standard deviation (mean ± SD). For Table 1, differences among DNA extraction methods were analyzed by one-way analysis of variance (one-way ANOVA), followed by Duncan’s multiple range test for post hoc comparisons. For Table 5, differences among samples were analyzed separately within each product category and sampling time using one-way ANOVA followed by Duncan’s multiple range test. A value of *p* < 0.05 was considered statistically significant. All experiments were performed in triplicate.

## 3. Results and Discussion

### 3.1. DNA Extraction

The performance of the tested DNA extraction methods, including heat-based methods (heating shaker, water bath, and microwave), ethanol precipitation, and a commercial kit, was evaluated in terms of DNA concentration and purity (Table 1). DNA purity was assessed using A_260_/A_280_ and A_260_/A_230_ ratios, which are commonly used indicators of protein, phenolic, and other contaminant residues in nucleic acid samples [8]. Among the tested methods, the commercial kit yielded the highest DNA concentration (64.19 ng/μL), followed by ethanol precipitation (35.26 ng/μL). Heat-based methods resulted in comparatively lower DNA yields, with concentrations ranging from 17.55 to 25.43 ng/μL for the heating shaker (99 °C), 23.17 to 27.42 ng/μL for the water bath (100 °C), and 15.27 to 17.49 ng/μL for microwave treatment (800 W). In terms of DNA purity, the commercial kit exhibited the highest A_260_/A_280_ ratio (1.97), indicating minimal protein contamination, followed by ethanol precipitation (1.73). In contrast, all heat-based methods showed lower A_260_/A_280_ values (1.42–1.70), suggesting the presence of residual proteins or phenolic compounds. A similar trend was observed for A_260_/A_230_ ratios, where the commercial kit (1.64) outperformed heat-based methods (0.78–1.39), indicating more effective removal of organic contaminants. The lower purity observed in heat-based methods may be attributed to the absence of purification steps, resulting in co-extraction of cellular debris and inhibitory substances. Significant differences among extraction methods are indicated in Table 1. Although heat treatment is known to disrupt cell membranes and release DNA [6], the lack of chemical or column-based purification limits its effectiveness in removing contaminants.

In contrast, the ethanol precipitation method improved DNA purity through the use of enzymatic lysis buffer and alcohol-mediated DNA precipitation, which facilitated the removal of impurities. However, the commercial kit demonstrated superior performance due to its silica membrane-based purification system, which selectively binds DNA while allowing contaminants such as salts and proteins to be washed away. Similar trends have been reported with previous studies indicating that efficient removal of PCR inhibitors is critical when analyzing food samples, as complex matrices may contain compounds that interfere with DNA amplification [12]. Therefore, although heat-based methods offer advantages in terms of simplicity, speed, and cost, the commercial kit was considered the most suitable method for downstream real-time PCR analysis in this study.

### 3.2. Comparison and Selection of DNA Extraction Methods

The primary purpose of DNA extraction is to obtain templates suitable for downstream molecular analyses; however, different extraction methods may significantly affect PCR amplification efficiency [13]. Therefore, the amplifiability of DNA obtained from different extraction methods was further evaluated using real-time PCR followed by agarose gel electrophoresis. Both the heating shaker (10–30 min) and water bath (10 min) methods produced detectable bands at bacterial concentrations ranging from 4 to 8 Log CFU/mL, whereas no bands were observed below 3 Log CFU/mL. For extended water bath treatments (20 and 30 min), detectable amplification was observed only at higher concentrations (5–8 Log CFU/mL), indicating reduced amplification efficiency (Figures S1 and S2). Microwave-based extraction demonstrated improved performance, with detectable amplification down to 3 Log CFU/mL for 30 s treatment, while 10 s and 60 s treatments showed detection limits of 4 Log CFU/mL. For chemical-based methods, ethanol precipitation showed a detection limit of 3 Log CFU/mL, whereas the commercial kit achieved the lowest detection limit of 2 Log CFU/mL (Figures S3 and S4). These results indicate that the commercial kit provided the highest DNA extraction efficiency and PCR amplifiability, followed by microwave treatment (30 s) and ethanol precipitation. A summary of detection limits for all extraction methods is presented in Table 2. The superior performance of the commercial kit can be attributed to its effective removal of PCR inhibitors and higher DNA purity, which are critical factors for successful amplification. In contrast, heat-based methods may co-extract inhibitory substances, thereby reducing PCR sensitivity.

Similar trends have been reported by Li et al. [5], who demonstrated that DNA extraction efficiency and pre-treatment methods significantly influence PCR detection limits. In their study, filtration-based enrichment improved detection sensitivity compared to centrifugation, and commercial kit-based extraction yielded higher DNA purity and lower detection limits than heat-based methods. Similar trends were observed in the present study, further confirming that appropriate sample preparation and DNA purification are essential for enhancing PCR detection performance. Considering the overall performance, including DNA quality, detection sensitivity, processing time, and cost, the commercial kit and microwave treatment (30 s) were selected for subsequent experiments. The commercial kit was chosen for its high extraction efficiency and reliability, whereas microwave treatment was selected as a rapid and cost-effective alternative for preliminary screening.

### 3.3. Optimization of PCR Conditions

#### 3.3.1. Primer Selection

**Figure 1:**
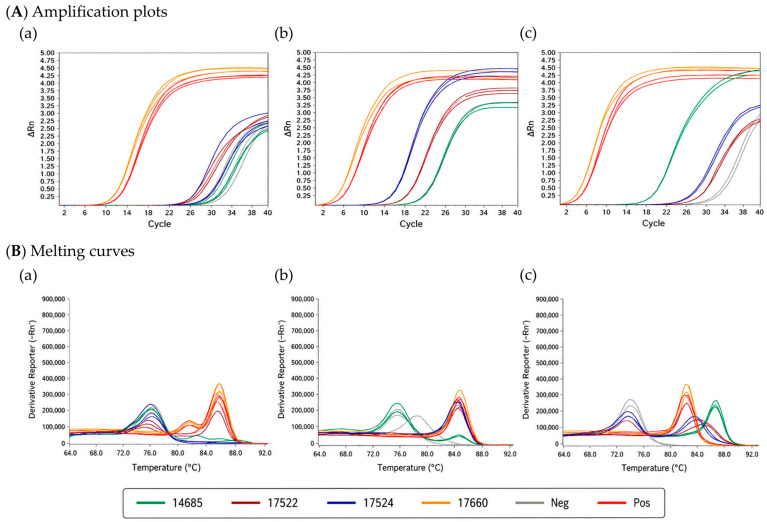
Comparison of the specificity of three primer sets for *Alicyclobacillus* spp.: A1-92-3 (**a**), *NAtb* (**b**), and *vdcC* (**c**).

Three primer sets (A1-92-3, *NAtb*, and *vdcC*) were evaluated for their performance in detecting *A. acidoterrestris*. The evaluation was conducted using four *Alicyclobacillus* strains, including *A. acidoterrestris* BCRC 17660, *A. cycloheptanicus* BCRC 17522, *A. hesperidum* BCRC 17524, and *A. acidocaldarius* BCRC 14685, as shown in Figure 1. All primer sets produced amplification curves due to the use of SYBR Green dye, which non-specifically binds to double-stranded DNA. Therefore, melting curve analysis was applied to assess primer specificity. For the A1-92-3 primer set, although amplification was observed for all tested strains, the melting temperatures (Tm) of *A. acidoterrestris* and *A. cycloheptanicus* were highly similar, making it difficult to distinguish between target and non-target species. Similarly, the *NAtb* primer set showed overlapping melting curves among all tested strains, indicating poor discriminatory capability despite strong amplification signals.

In contrast, the *vdcC* primer set demonstrated clear differentiation between *A. acidoterrestris* and other *Alicyclobacillus* species. The target strain exhibited a distinct melting temperature (~85.3 °C), whereas non-target strains showed significantly different Tm values. This allowed for reliable discrimination based on melting curve analysis. Similar trends have been reported with previous studies. Wang et al. [9] reported that the *vdcC* gene contains species-specific regions suitable for selective detection of *A. acidoterrestris*. In addition, Li et al. [5] demonstrated that primer design targeting unique conserved regions is critical for achieving high specificity. Based on these results, the *vdcC* primer set was selected for subsequent analyses due to its superior specificity and reliable discrimination capability.

#### 3.3.2. Reagents Selection

Two commercial real-time PCR master mixes, TB Green Premix Ex Taq II and KAPA SYBR FAST qPCR Master Mix, were evaluated for their performance in detecting *A. acidoterrestris*, as shown in Figures S5 and S6. Both reagents produced amplification signals due to the use of SYBR Green chemistry. However, differences were observed in their ability to discriminate target and non-target strains based on melting curve analysis. The TB Green reagent showed overlapping melting peaks among all tested strains, making it difficult to distinguish *A. acidoterrestris* from other *Alicyclobacillus* species.

In contrast, the KAPA SYBR FAST reagent provided distinct melting temperature (Tm) profiles for each strain, allowing for clear differentiation of *A. acidoterrestris* from non-target species. These results indicate that the choice of master mix can influence assay specificity, particularly in SYBR Green-based systems where melting curve analysis is critical. The KAPA SYBR FAST qPCR Master Mix was selected for subsequent analyses due to its superior discriminatory performance.

### 3.4. Specificity

**Figure 2:**
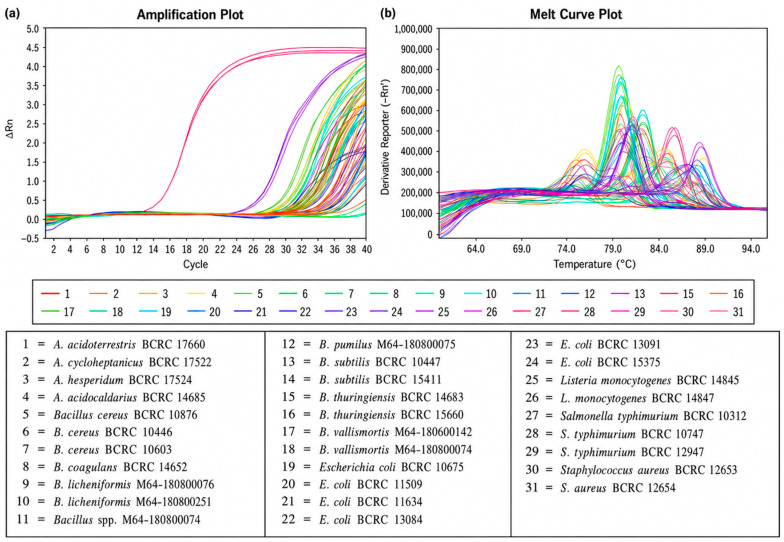
Specificity test of *vdcC* gene using 31 strains. (**a**) *A. acidoterrestris* BCRC 17660 produced a fluorescence amplification curve at Ct value 12.935. (**b**) Melting curve. The Tm value of *A. acidoterrestris* BCRC 17660 is 85.17.

The specificity of the real-time PCR assay targeting the *vdcC* gene was evaluated using a total of 31 bacterial strains, including one *A. acidoterrestris* strain, three non-target *Alicyclobacillus* species, and 27 other common foodborne microorganisms. Genomic DNA was extracted using the DNeasy Blood & Tissue Kit and analyzed using KAPA SYBR FAST qPCR Master Mix on a QuantStudio 5 Real-Time PCR System. As shown in Figure 2, amplification signals were observed not only for *A. acidoterrestris* but also for several non-target strains. However, further analysis of melting curves (Figure 2b) revealed that only *A. acidoterrestris* produced a specific amplicon with a characteristic melting temperature (Tm) of 85.17 °C. In contrast, non-target strains exhibited different melting profiles, indicating non-specific amplification. These results demonstrate that although non-specific fluorescence signals may occur in SYBR Green-based assays, the combination of amplification curves and melting curve analysis enables accurate discrimination of *A. acidoterrestris*. Therefore, the *vdcC* primer set showed high specificity for target detection.

The specificity observed in this study is consistent with previous reports. For example, Wang et al. [9] demonstrated that *vdcC*-targeting primers selectively amplified *A. acidoterrestris* without cross-reactivity with other *Alicyclobacillus* or *Bacillus* species. Similar findings were obtained in the present study, further confirming the reliability of *vdcC* as a molecular marker for specific detection of *A. acidoterrestris*. Although probe-based systems such as TaqMan may provide higher analytical specificity, the SYBR Green format was intentionally selected in this study because of its lower cost, simpler assay design, and broader accessibility in routine food testing laboratories. Using this format, melting curve analysis was essential to improve discrimination between target and non-target signals. Nevertheless, the specificity assessment would be further strengthened by including a broader range of juice-associated *Alicyclobacillus* species, such as *A. pomorum*, *A. herbarius*, and *A. mali*, which should be considered in future studies.

### 3.5. Limit of Detection

**Figure 3:**
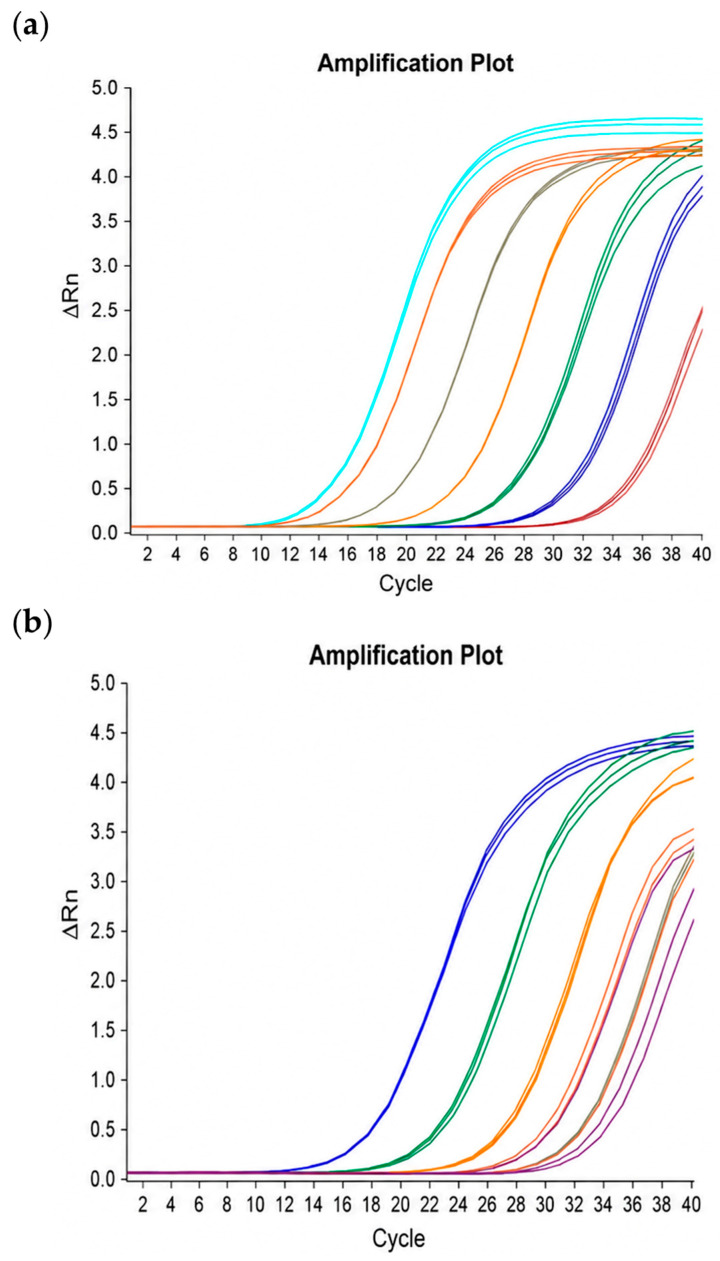
Detection limit of the real-time PCR assay using DNA extracted with the commercial kit (**a**) and microwave treatment (**b**). From left to right, the bacterial concentrations ranged from 8 to 2 Log CFU/mL in panel (**a**) and from 8 to 3 Log CFU/mL in panel (**b**).

The limit of detection (LOD) of the real-time PCR assay targeting the *vdcC* gene was evaluated using *A. acidoterrestris* BCRC 17660 as the reference strain. The bacterial culture (approximately 8 Log CFU/mL) was serially diluted (10-fold), and DNA was extracted from each dilution using either a commercial kit (DNeasy Blood & Tissue Kit) or microwave treatment (30 s). Real-time PCR was performed using KAPA SYBR FAST qPCR Master Mix on a QuantStudio 5 Real-Time PCR System. As shown in Figure 3, amplification signals were detected in samples with concentrations ranging from 2 to 8 Log CFU/mL when using the commercial kit, whereas no amplification was observed at 1 Log CFU/mL. It should be noted that this LOD represents analytical sensitivity determined using serially diluted culture suspensions rather than a formal matrix-based LOD.

In contrast, microwave-based extraction (30 s) yielded detectable signals at concentrations from 3 to 8 Log CFU/mL, with no amplification observed at 1–2 Log CFU/mL. These results indicate that the LOD of the assay was 2 Log CFU/mL when DNA was extracted using the commercial kit, and 3 Log CFU/mL when using microwave treatment. The improved sensitivity observed with the commercial kit is likely due to its higher DNA purity and more effective removal of PCR inhibitors, which enhances amplification efficiency.

The LOD obtained in this study is comparable to or slightly better than previously reported values. Wang et al. [9] reported a detection limit of 2.41 Log CFU/mL for *A. acidoterrestris* in both sterile water and apple juice using a real-time PCR assay. The slightly lower detection limit achieved in the present study (2 Log CFU/mL) may be attributed to optimized DNA extraction and amplification conditions. In addition, studies targeting other foodborne pathogens, such as Huang et al. [14], have demonstrated that real-time PCR can achieve high sensitivity under optimized conditions. The findings of the present study further support the applicability of real-time PCR as a rapid and sensitive tool for detecting *A. acidoterrestris* in food-related samples.

Because the sensitivity analysis was performed using a single reference strain, future studies should include additional *A. acidoterrestris* isolates from different sources to further evaluate inter-strain variation in assay performance. To better position the present study within the existing literature, a comparison of previously reported PCR-based methods for the detection of *Alicyclobacillus* spp. in juice-related matrices is summarized in Table 3.

As shown in Table 3, previously reported PCR-based methods range from conventional PCR and fingerprinting approaches to real-time PCR, immunomagnetic separation-coupled PCR, and sequencing-based confirmation. Compared with these approaches, the present study focuses on practical workflow optimization for fruit juice applications by systematically evaluating DNA extraction methods, primer selection, reagent performance, matrix applicability, and interpretation of PCR positivity in relation to guaiacol analysis.

Accordingly, the contribution of this work lies not in the introduction of a new molecular target, but in the optimization and application of an accessible real-time PCR workflow for juice quality monitoring.

### 3.6. Evaluation of Juice Matrix Effects

To evaluate the applicability of the developed real-time PCR method in complex food systems, matrix spiking experiments were conducted using three commonly consumed juice products, including orange juice, apple juice, and grape juice. *A. acidoterrestris* BCRC 17660 was cultured and serially diluted (10-fold), and aliquots were spiked into each juice matrix. DNA was extracted using either microwave treatment (30 s) or a commercial kit, followed by real-time PCR analysis. Comparable amplification trends were also observed in apple and grape juice matrices, whereas orange juice is presented here as a representative example.

**Figure 4:**
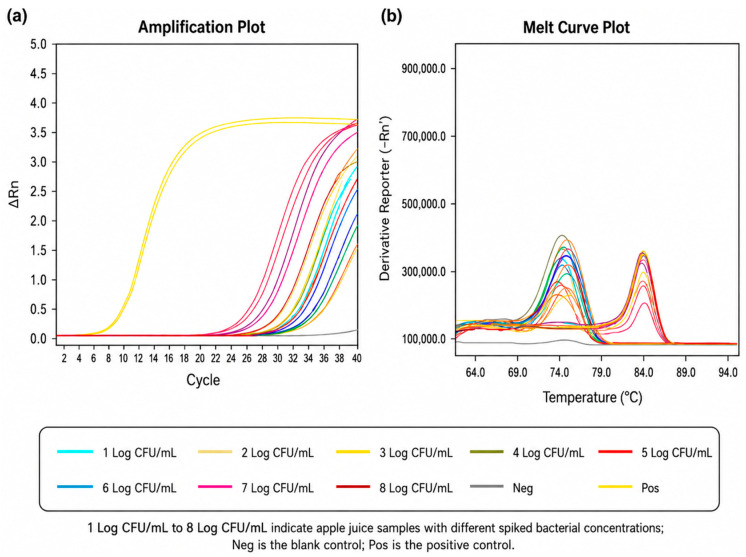
Orange juice spike test using microwave for 30 s to extract DNA. (**a**) Amplification plot. The Ct value range of spiked bacterial concentrations from 8 to 1 Log CFU/mL was 28.147 to 33.570. The Ct value of the positive control was 13.147, and the Ct value of the blank control was 34.268. (**b**) Melting curve. The Tm value of the positive control group was 85.01. The orange juice supplemented with bacterial solution at concentrations of 6 to 8 Log CFU/mL had the same Tm value.

For microwave-based extraction, amplification signals were observed across all tested concentrations and matrices (Figure 4). However, due to the non-specific nature of SYBR Green detection, amplification curves alone were insufficient for target identification. Melting curve analysis revealed that characteristic Tm values (~85.0 °C) corresponding to *A. acidoterrestris* were detected within specific concentration ranges. Specifically, consistent Tm values were observed at 8–6 Log CFU/mL in orange juice. These results indicate that matrix components did not significantly interfere with target detection at higher bacterial concentrations, although sensitivity was limited at lower concentrations. For the commercial kit-based extraction, amplification signals were also detected across all matrices (Figure 4). This indicates improved sensitivity and robustness compared to microwave-based extraction.

The assay successfully detected *A. acidoterrestris* in the tested juice matrices, with the commercial kit showing higher sensitivity and microwave extraction serving as a rapid alternative. Similar trends have been reported with previous studies. Li et al. [5] reported a strong correlation between bacterial concentration and Ct values in spiked apple juice samples, indicating reliable detection performance. In addition, Cai et al. [25] demonstrated that appropriate sample pre-treatment could effectively reduce matrix interference and improve detection sensitivity. The results of the present study further confirm that matrix effects can be minimized through suitable DNA extraction methods, enabling accurate detection of *A. acidoterrestris* in juice products. An internal amplification control was not included in the present assay; therefore, PCR inhibition in complex juice matrices cannot be completely excluded and should be addressed in future assay refinement.

### 3.7. Market Survey

#### 3.7.1. Detection of *A. acidoterrestris* by Real-Time PCR

**Figure 5:**
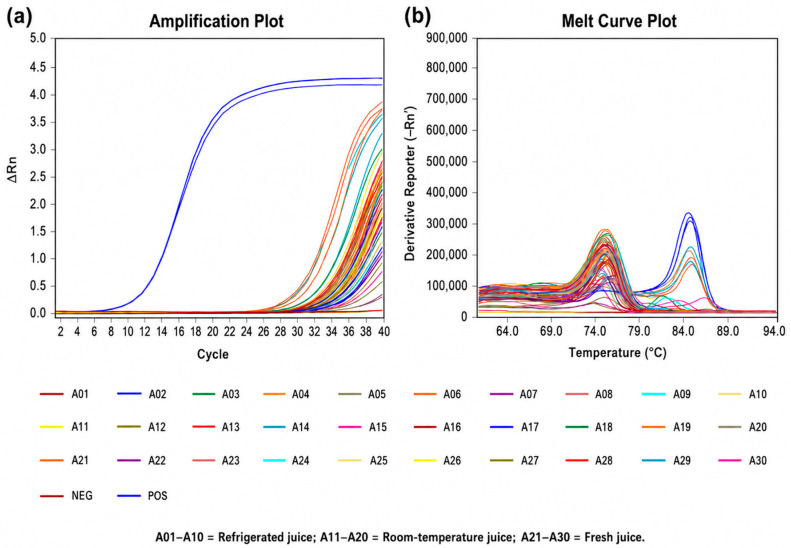
Real-time PCR results of 30 commercial fruit juice samples on culture day 0. (**a**) Amplification plot. The Ct value of the positive control was 12.292, the Ct value of the blank control was 34.617, and the average Ct value of the 30 samples ranged from 29.558 to 35.177. (**b**) Melting curve: the Tm value of the positive control group was 85.235, the Tm value of juice sample A21 was 85.310, the Tm value of A29 was 85.330, and the Tm values of the remaining 28 samples ranged from 59.986 to 84.759.

**Figure 6:**
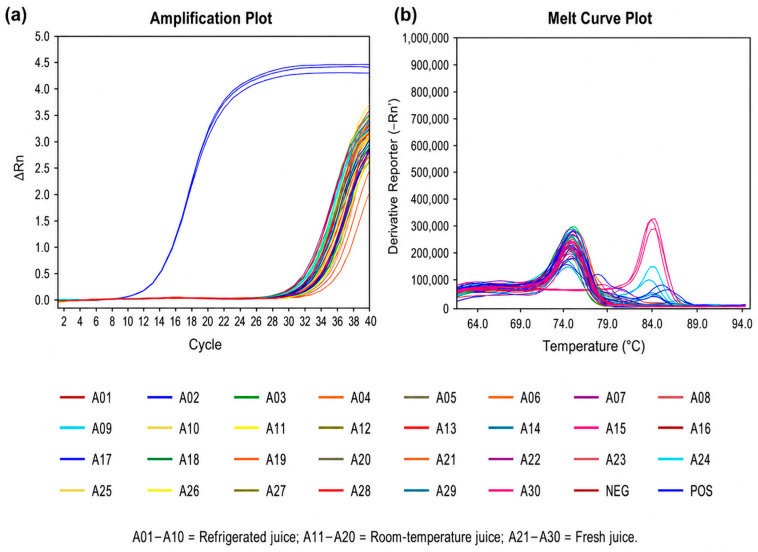
Real-time PCR results of 30 commercial fruit juice samples on culture day 1. (**a**) Amplification plot. The Ct value of the positive control was 14.975, the Ct value of the blank control was 33.074, and the average Ct value of the 30 samples ranges from 32.368 to 34.615. (**b**) Melting curve: the Tm value of the positive control group was 85.137, the Tm value of juice sample A21 was 85.169, the Tm value of A29 was 84.994, and the Tm values of the remaining 28 samples ranged from 75.795 to 76.945.

A total of 30 commercial juice samples, including reconstituted juice, commercially sterilized juice, and freshly prepared juice, were collected from supermarkets, traditional markets, beverage shops, and vending machines. DNA was extracted using the DNeasy Blood & Tissue Kit, followed by real-time PCR analysis. Amplification signals were observed in all samples, including negative controls, indicating the presence of non-specific fluorescence signals inherent to SYBR Green-based detection (Figure 5 and Figure 6). Therefore, melting curve analysis was employed for accurate identification. As shown in Figure 5b and Figure 6b, only two samples (A21 and A29) exhibited melting temperatures consistent with the positive control (~85 °C), while the remaining samples showed distinct Tm profiles. Sequencing of PCR products from these two samples, followed by comparison with the NCBI GenBank database, confirmed the presence of *A. acidoterrestris* (Table 4).

Notably, no bacterial colonies were observed using conventional culture methods at both 0 and 1 day of incubation, suggesting that the detected DNA originated from non-viable cells. In addition to DNA from non-viable cells, SYBR Green-based PCR may also detect free DNA or extracellular DNA present in the sample, which should be considered when interpreting PCR-positive but culture-negative results. The comparison between day 0 and day 1 results further provides insight into whether the detected *A. acidoterrestris* represents viable cells capable of growth or residual DNA from non-viable cells. These findings suggest that real-time PCR can detect target DNA derived from viable cells, non-viable cells, or extracellular DNA, thereby providing higher analytical sensitivity than culture-based methods. The positive results in samples A21 and A29 may be associated with cross-contamination during processing or inadequate cleaning of equipment.

These results are consistent with previous studies. Cai et al. [25] reported high sensitivity and accuracy of real-time PCR in detecting *A. acidoterrestris* in fruit juice samples. Similarly, Shayanfar et al. [29] demonstrated that even low levels of *A. acidoterrestris* could be detected within 24 h of incubation using real-time PCR. These findings should be regarded as preliminary observations from a limited set of commercial samples rather than as evidence of market prevalence.

#### 3.7.2. Determination of Guaiacol Content

Guaiacol content was analyzed by HPLC, with a retention time of approximately 4.44 min. Among the 30 commercial juice samples, guaiacol was detected in several samples at day 0, with concentrations ranging from 76.82 to 251.67 μg/mL. After 1 day of incubation, guaiacol was detected in only four samples, with concentrations ranging from 74.89 to 81.99 μg/mL. Although *A. acidoterrestris* DNA was detected in samples A21 and A29, no corresponding increase in guaiacol concentration was observed after incubation, suggesting that the detected cells were likely non-viable. Significant differences among samples within each product category are shown in Table 5. The reduced guaiacol levels after enrichment may also be partially explained by dilution during sample preparation. These results suggest that molecular detection alone may overestimate spoilage risk when interpreted without considering viability and metabolic activity. Therefore, combining real-time PCR with guaiacol analysis may provide a more comprehensive evaluation of juice quality. Under the tested conditions, PCR positivity alone was not sufficient to infer active spoilage. Guaiacol formation requires not only the presence of target DNA, but also viable cells, active metabolism, and suitable environmental conditions.

**Table 5:** Guaiacol concentration of commercial juice samples by product type.

Type	Sample No.	Culture Day 0 (μg/mL)	Culture Day 1 (μg/mL)
Refrigeration	A01	207.96 ± 0.75 ^a^	76.53 ± 0.13 ^a^
	A02	203.26 ± 0.84 ^b^	74.89 ± 0.12 ^b^
	A03	135.98 ± 0.36 ^c^	N.D.
	A04	N.D.	N.D.
	A05	N.D.	N.D.
	A06	108.72 ± 0.52 ^d^	N.D.
	A07	N.D.	N.D.
	A08	86.94 ± 2.74 ^e^	N.D.
	A09	N.D.	N.D.
	A10	N.D.	N.D.
Room temperature	A11	115.43 ± 0.19 ^a^	N.D.
	A12	89.59 ± 0.04 ^b^	N.D.
	A13	93.91± 4.86 ^b^	N.D.
	A14	N.D.	N.D.
	A15	N.D.	N.D.
	A16	N.D.	N.D.
	A17	117.50 ± 15.99 ^a^	N.D.
	A18	N.D.	N.D.
	A19	N.D.	N.D.
	A20	N.D.	N.D.
Fresh	A21	251.67 ± 0.87 ^a^	81.99 ± 0.17 ^a^
	A22	144.00 ± 1.42 ^b^	N.D.
	A23	215.46 ± 7.70 ^b^	76.88 ± 0.16 ^b^
	A24	82.81 ± 0.23 ^f^	N.D.
	A25	76.82 ± 0.20 ^g^	N.D.
	A26	119.46 ± 4.62 ^d^	N.D.
	A27	N.D.	N.D.
	A28	119.33 ± 0.15 ^d^	N.D.
	A29	107.39 ± 0.41 ^e^	N.D.
	A30	N.D.	N.D.

N.D.: Not Detected. Values in the same column with different letters are significantly different (*p* < 0.05).

Previous studies have indicated that guaiacol production is influenced by bacterial viability and incubation time. Integrating real-time PCR with guaiacol analysis may provide a more comprehensive and practical approach for evaluating juice quality. Bahçeci and Acar [30] reported that detectable guaiacol production requires sufficient incubation time and bacterial growth, even when spores are present. Although guaiacol concentrations detected in this study exceeded reported sensory thresholds in water [31], the complex composition of juice matrices, including sugars and aroma compounds, may mask off-flavors. Furthermore, the persistence of guaiacol in commercial juice products may be attributed to the thermal resistance of *A. acidoterrestris* spores, which can survive pasteurization and subsequently germinate during storage [32]. In freshly prepared juices, contamination may originate from raw materials or biofilm formation on processing equipment [33]. These observations should therefore be interpreted cautiously, particularly because the commercial samples originated from heterogeneous sources and processing conditions.

## 4. Conclusions

This study demonstrates that a SYBR Green-based real-time PCR assay targeting the *vdcC* gene can be effectively used for the detection of *A. acidoterrestris* in fruit juice. The commercial DNA extraction kit provided superior performance, achieving a detection limit of 2 Log CFU/mL, while microwave-based extraction offered a rapid alternative with slightly lower sensitivity. The use of the *vdcC* primer set, together with melting curve analysis, enabled reliable discrimination of *A. acidoterrestris* from closely related species. The commercial sample survey was intended as a preliminary application test of the assay and should not be interpreted as a prevalence study. Taken together, these findings support the use of this method as a practical workflow for juice quality monitoring rather than as a newly designed molecular target system.

## Figures and Tables

**Table 1:** DNA concentration and purity of different DNA extraction methods.

Method			DNA Conc. (ng/μL)	DNA Purity A_260_/A_280_	DNA Purity A_260_/A_230_
Heat	Thermal shaker (99 °C)	10 min	25.43 ± 0.31 ^e^	1.52 ± 0.03 ^gh^	0.96 ± 0.00 ^f^
		20 min	18.30 ± 0.08 ^g^	1.55 ± 0.02 ^fg^	1.18 ± 0.04 ^de^
		30 min	17.55 ± 0.31 ^h^	1.65 ± 0.02 ^cd^	1.23 ± 0.03 ^d^
	Water bath (100 °C)	10 min	27.42 ± 0.03 ^c^	1.46 ± 0.02 ^ij^	0.96 ± 0.01 ^f^
		20 min	23.17 ± 0.08 ^f^	1.62 ± 0.06 ^de^	1.29 ± 0.06 ^c^
		30 min	26.28 ± 0.03 ^d^	1.70 ± 0.04 ^bc^	1.15 ± 0.00 ^e^
	Microwave (800 W)	10 s	15.27 ± 0.12 ^i^	1.47 ± 0.02 ^hi^	1.39 ± 0.02 ^b^
		30 s	17.49 ± 0.05 ^h^	1.59 ± 0.03 ^ef^	1.34 ± 0.06 ^bc^
		1 min	17.48 ± 0.06 ^h^	1.42 ± 0.02 ^j^	0.78 ± 0.01 ^g^
Ethanol			35.26 ± 0.07 ^b^	1.73 ± 0.02 ^b^	0.18 ± 0.00 ^h^
Commercial Kit			64.19 ± 0.03 ^a^	1.97 ± 0.02 ^a^	1.64 ± 0.00 ^a^

Values in the same column with different letters are significantly different (*p* < 0.05).

**Table 2:** Detection limits of different DNA extraction methods.

Method			Bacterial Counts (CFU/mL)							
			1 Log	2 Log	3 Log	4 Log	5 Log	6 Log	7 Log	8 Log
Heat	Thermal shaker (99 °C)	10 min	−	−	−	+	+	+	+	+
		20 min	−	−	−	+	+	+	+	+
		30 min	−	−	−	+	+	+	+	+
	Water bath (100 °C)	10 min	−	−	−	+	+	+	+	+
		20 min	−	−	−	−	+	+	+	+
		30 min	−	−	−	−	+	+	+	+
	Microwave (800 W)	10 s	−	−	−	+	+	+	+	+
		30 s	−	−	+	+	+	+	+	+
		1 min	−	−	−	+	+	+	+	+
Ethanol			−	−	+	+	+	+	+	+
Commercial Kit			−	+	+	+	+	+	+	+

+: detected; −: not detected.

**Table 3:** Comparison of published PCR-based methods for the detection of *A. acidoterrestris* in juice-related matrices.

Method	Representative Matrices or Sources	Main Application	Suitability for Routine Industry Use	Main Limitations	Reference(s)
Conventional PCR	Juice blends, mango juice, fruit concentrates, soils	Species detection/confirmation	Moderate	Endpoint detection only; no Ct-based information; lower practical value for rapid routine screening	[15,16,17,18]
PCR-based typing methods (RAPD-PCR, ERIC-PCR, PCR-RFLP)	Orchard soil, fruit surfaces, processing environments, concentrated juices, orange juice	Strain differentiation and source tracking	Low	Lower reproducibility or greater procedural complexity; not ideal for routine industrial screening	[19,20,21]
PCR-DGGE	Fruit juices and fruit juice blends	Detection and differentiation of target species, including guaiacol-producing strains	Low	Technically complex; limited practicality for routine quality control	[22]
Real-time PCR	Orange juice, apple juice, sports drink, lemonade, flavored drinks, acid buffer	Rapid detection and semi-quantitative analysis	High	Requires specialized instrumentation; assay specificity depends on chemistry and primer/probe design	[23,24]
IMS-PCR/IMS-RT-PCR	Apple juice, kiwi juice, orchard and production-line samples	Improved detection in complex matrices	Moderate to high	More labor-intensive and costly than direct PCR-based workflows	[10,25,26]
qPCR	Apple juice	Quantitative or semi-quantitative detection	High	Requires calibration and validation for matrix effects	[5]
16S rRNA sequencing	Juice-, fruit-, soil-, and environment-related isolates	Confirmatory identification	Low	Time-consuming and less suitable for routine industrial monitoring	[27]

Note: Information summarized from the review by Sourri et al. [28] on detection methods for *Alicyclobacillus* spp. in fruit juice and related matrices.

**Table 4:** Real-time PCR product BLAST results of positive juice samples.

Culture Time	Sample	BLAST Result
0 day	A21	*Alicyclobacillus acidoterrestris* strain 41 *VdcB* (*vdcB*), *VdcC* (*vdcC*), and *VdcD* (*vdcD*) genes, complete cds
	A29	*Alicyclobacillus acidoterrestris* strain 41 *VdcB* (*vdcB*), *VdcC* (*vdcC*), and *VdcD* (*vdcD*) genes, complete cds
	Positive	*Alicyclobacillus acidoterrestris* strain 41 *VdcB* (*vdcB*), *VdcC* (*vdcC*), and *VdcD* (*vdcD*) genes, complete cds
1 day	A21	*Alicyclobacillus acidoterrestris* strain 41 *VdcB* (*vdcB*), *VdcC* (*vdcC*), and *VdcD* (*vdcD*) genes, complete cds
	A29	*Alicyclobacillus acidoterrestris* strain 41 *VdcB* (*vdcB*), *VdcC* (*vdcC*), and *VdcD* (*vdcD*) genes, complete cds
	Positive	*Alicyclobacillus acidoterrestris* strain 41 *VdcB* (*vdcB*), *VdcC* (*vdcC*), and *VdcD* (*vdcD*) genes, complete cds

## Data Availability

The original contributions presented in the study are included in the article/Supplementary Materials; further inquiries can be directed to the corresponding author.

## Supplementary Materials

The following supporting information can be downloaded at: https://www.mdpi.com/article/10.3390/foods15101672/s1, Figure S1: Gel electrophoresis of DNA extracted from different concentrations of *A. acidoterrestris* bacterial suspensions heated on a heating shaker (99°C, 500 rpm) for (a) 10 min, (b) 20 min, and (c) 30 min. Figure S2: Gel electrophoresis of DNA extracted from *A. acidoterrestris* bacterial suspensions of different concentrations heated in a water bath (100 °C) for (a) 10 min, (b) 20 min, and (c) 30 min. Figure S3: Gel electrophoresis of DNA extracted from *A. acidoterrestris* bacterial suspensions of different concentrations heated by microwave (800 W) for (a) 10 s, (b) 30 s, and (c) 60 s. Figure S4: Gel electrophoresis of DNA extracted from *A. acidoterrestris* bacterial suspensions of different concentrations using (a) the alcohol extraction method and (b) the commercially available kit method. Figure S5: Test results of *vdcC* primers using TAKARA TB GREEN reagent. (a) Amplification plot. (b) Melting curves. Figure S6: Test results of *vdcC* primers using KAPA SYBR GREEN reagent.
