# Supplementary material for: Real-Time PCR Detection of Alicyclobacillus acidoterrestris in Fruit Juice: Method Validation and Implications for Guaiacol-Related Spoilage
Source: Foods. 2026 May 11;15(10):1672. doi: 10.3390/foods15101672 (PMC13206414; doi:10.3390/foods15101672)
Supplement: Supplementary file 1 [file foods-15-01672-s001.zip › foods-4275358-supplementary.pdf]

Supplementary information

Figure S1. Gel electrophoresis of DNA extracted from different concentrations of *Alicyclobacillus acidoterrestris* bacterial suspensions heated on a heating shaker (99°C, 500 rpm) for (a) 10 min, (b) 20 min, and (c) 30 min. Lane 1-8: 1 Log CFU/mL, 2 Log CFU/mL, 3 Log CFU/mL, 4 Log CFU/mL, 5 Log CFU/mL, 6 Log CFU/mL, 7 Log CFU/mL and 8 Log CFU/mL, M: DNA marker (100 bp); PC: Postive control.

Figure S2. Gel electrophoresis of DNA extracted from *Alicyclobacillus acidoterrestris* bacterial suspensions of different concentrations heated in a water bath (100°C) for (a) 10 min, (b) 20 min, and (c) 30 min. Lane 1-8: 1 Log CFU/mL, 2 Log CFU/mL, 3 Log CFU/mL, 4 Log CFU/mL, 5 Log CFU/mL, 6 Log CFU/mL, 7 Log CFU/mL and 8 Log CFU/mL, M: DNA marker (100 bp); PC: Postive control.

Figure S3. Gel electrophoresis of DNA extracted from *Alicyclobacillus acidoterrestris* bacterial suspensions of different concentrations heated by microwaves (800W) for (a) 10 seconds, (b) 30 seconds, and (c) 60 seconds. Lane 1-8: 1 Log CFU/mL, 2 Log CFU/mL, 3 Log CFU/mL, 4 Log CFU/mL, 5 Log CFU/mL, 6 Log CFU/mL, 7 Log CFU/mL and 8 Log CFU/mL, M: DNA marker (100 bp); PC: Positive control.

Figure S4. Gel electrophoresis of DNA extracted from *Alicyclobacillus acidoterrestris* bacterial suspensions of different concentrations using (a) the alcohol extraction method and (b) the commercially available kit method. Lane 1-8: 1 Log CFU/mL, 2 Log CFU/mL, 3 Log CFU/mL, 4 Log CFU/mL, 5 Log CFU/mL, 6 Log CFU/mL, 7 Log CFU/mL and 8 Log CFU/mL, M: DNA marker (100 bp); PC: Positive control.

Figure S5. Test results of *vdcC* primer using TAKARA TB GREEN reagent. (a) Amplification plot. (b) Melting curves.

The Ct value of *A. acidoterrestris* BCRC 17660 was 15.561, the Ct value of *A. cycloheptanicus* BCRC 17522 was 35.266, the Ct value of *A. hesperidum* BCRC 17524 was 35.266, and the Ct value of *A. acidocaldarius* BCRC 14685 was 34.956. The Tm value of *A. acidoterrestris* BCRC 17660 was 87.304, the Tm value of *A. cycloheptanicus* BCRC 17522 was 87.012, the Tm value of *A. hesperidum* BCRC 17524 was 87.060, and the Tm value of *A. acidocaldarius* BCRC 14685 was 87.447.

Figure S6. Test results of *vdcC* primer using KAPA SYBR GREEN reagent.

(a) Amplification plot. (b) Melting curves.

The Ct value of *A. acidoterrestris* BCRC 17660 was 16.202, the Ct value of *A. cycloheptanicus* BCRC 17522 was 35.040, the Ct value of *A. hesperidum* BCRC 17524 was 34.502, and the Ct value of *A. acidocaldarius* BCRC 14685 was 28.446. The Tm value of *A. acidoterrestris* BCRC 17660 was 85.140, the Tm value of *A. cycloheptanicus* BCRC 17522 was 86.516, the Tm value of *A. hesperidum* BCRC 17524 was 86.326, and the Tm value of *A. acidocaldarius* BCRC 14685 was 88.177.

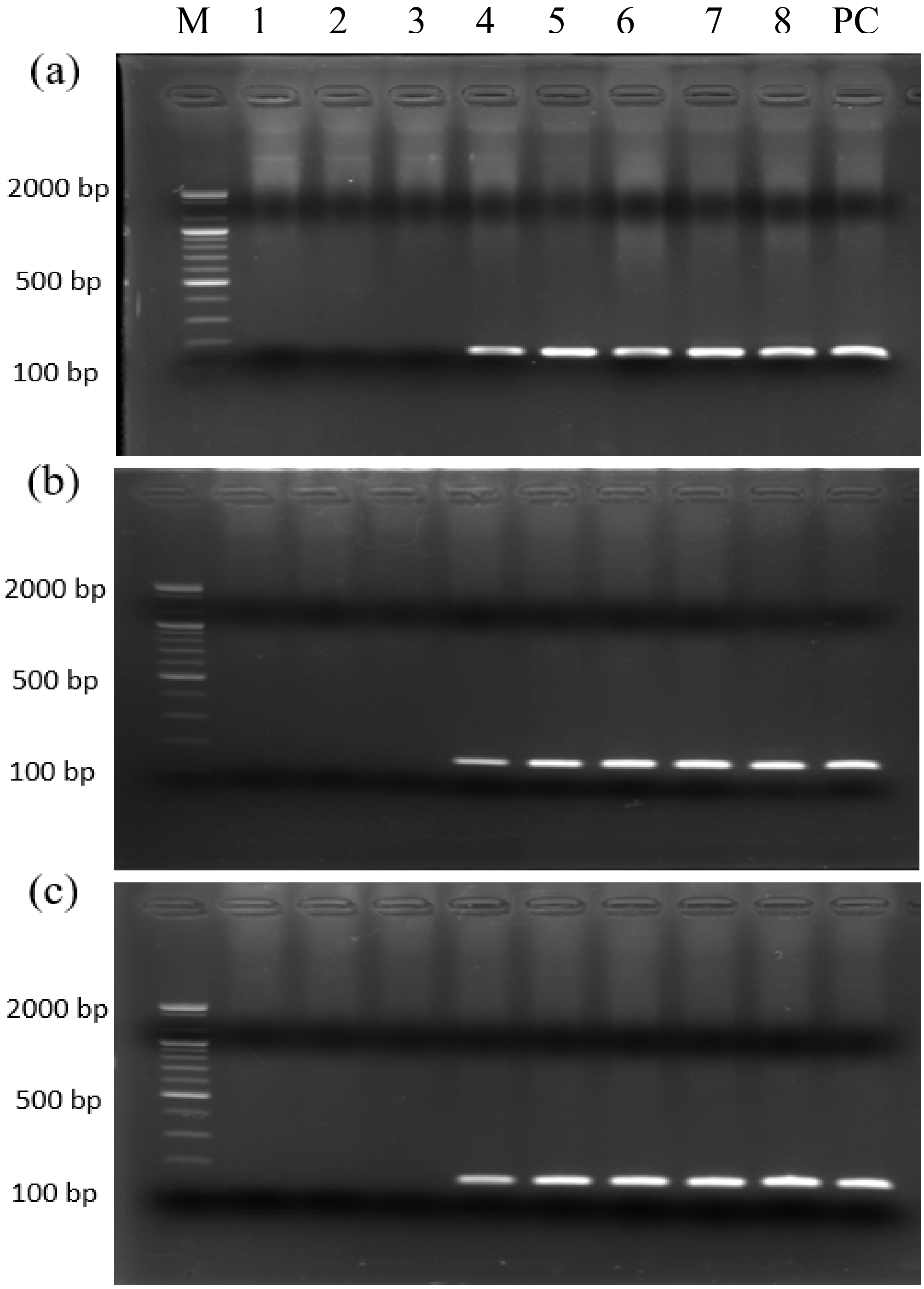

Figure S1. Gel electrophoresis of DNA extracted from different concentrations of *Alicyclobacillus acidoterrestris* bacterial suspensions heated on a heating shaker (99°C, 500 rpm) for (a) 10 min, (b) 20 min, and (c) 30 min. Lane 1-8: 1 Log CFU/mL, 2 Log CFU/mL, 3 Log CFU/mL, 4 Log CFU/mL, 5 Log CFU/mL, 6 Log CFU/mL, 7 Log CFU/mL and 8 Log CFU/mL, M: DNA marker (100 bp); PC: Postive control.

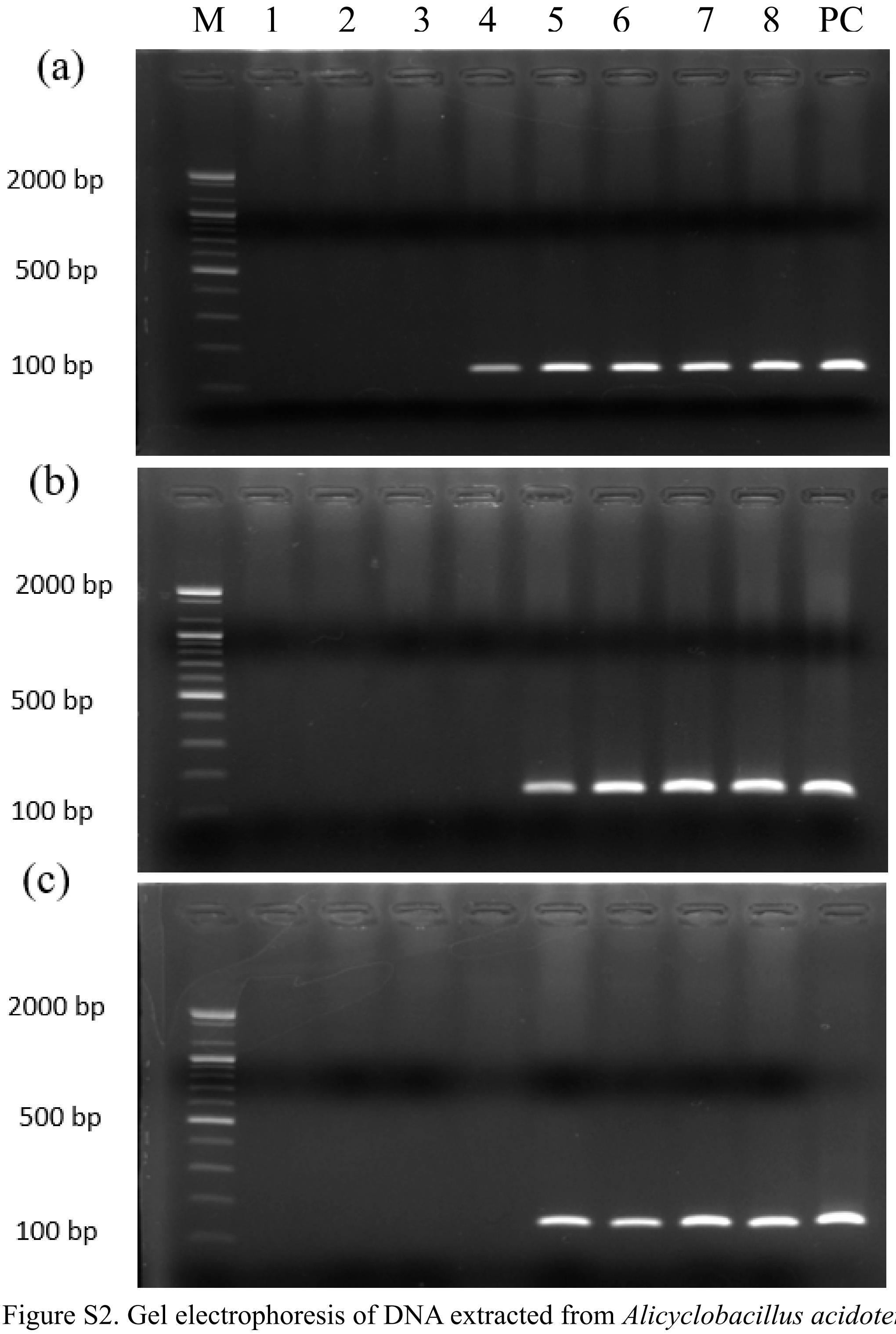

Figure S2. Gel electrophoresis of DNA extracted from *Alicyclobacillus acidoterrestris* bacterial suspensions of different concentrations heated in a water bath (100°C) for (a) 10 min, (b) 20 min, and (c) 30 min. Lane 1-8: 1 Log CFU/mL, 2 Log CFU/mL, 3 Log CFU/mL, 4 Log CFU/mL, 5 Log CFU/mL, 6 Log CFU/mL, 7 Log CFU/mL and 8 Log CFU/mL, M: DNA marker (100 bp); PC: Postive control.

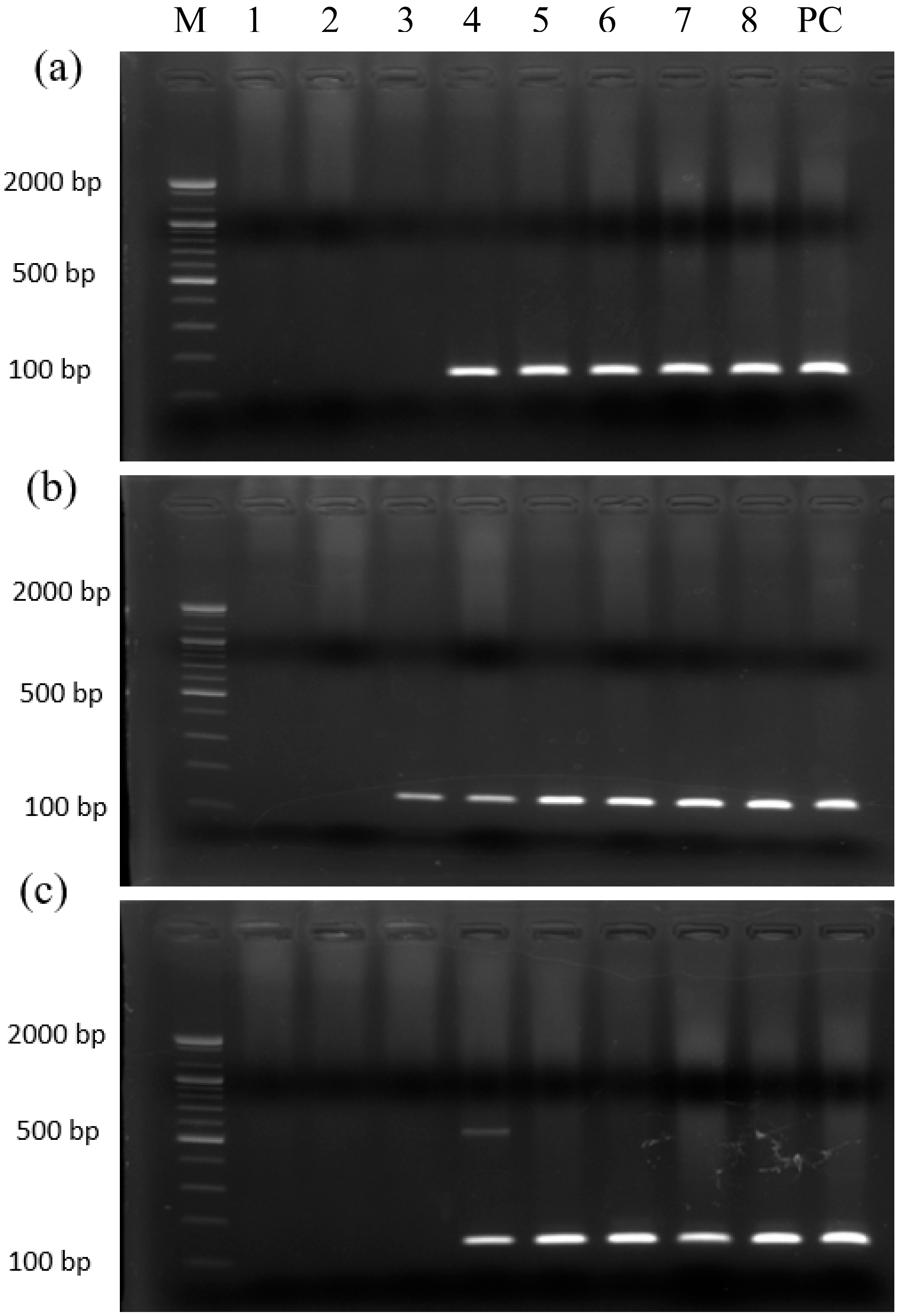

Figure S3. Gel electrophoresis of DNA extracted from *Alicyclobacillus acidoterrestris* bacterial suspensions of different concentrations heated by microwaves (800W) for (a) 10 seconds, (b) 30 seconds, and (c) 60 seconds. Lane 1-8: 1 Log CFU/mL, 2 Log CFU/mL, 3 Log CFU/mL, 4 Log CFU/mL, 5 Log CFU/mL, 6 Log CFU/mL, 7 Log CFU/mL and 8 Log CFU/mL, M: DNA marker (100 bp); PC: Positive control.

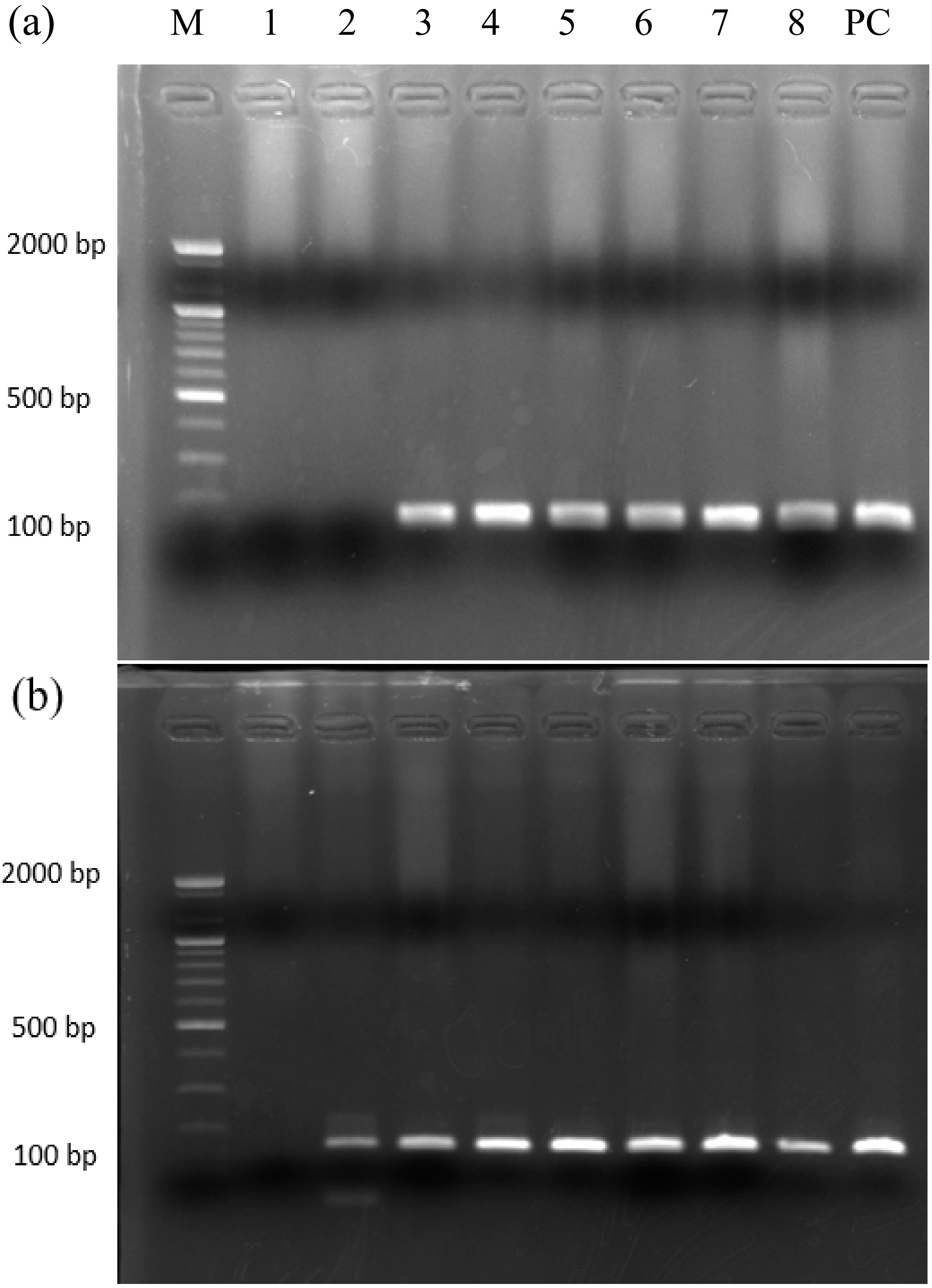

Figure S4. Gel electrophoresis of DNA extracted from *Alicyclobacillus acidoterrestris* bacterial suspensions of different concentrations using (a) the alcohol extraction method and (b) the commercially available kit method. Lane 1-8: 1 Log CFU/mL, 2 Log CFU/mL, 3 Log CFU/mL, 4 Log CFU/mL, 5 Log CFU/mL, 6 Log CFU/mL, 7 Log CFU/mL and 8 Log CFU/mL, M: DNA marker (100 bp); PC: Positive control.

(a)

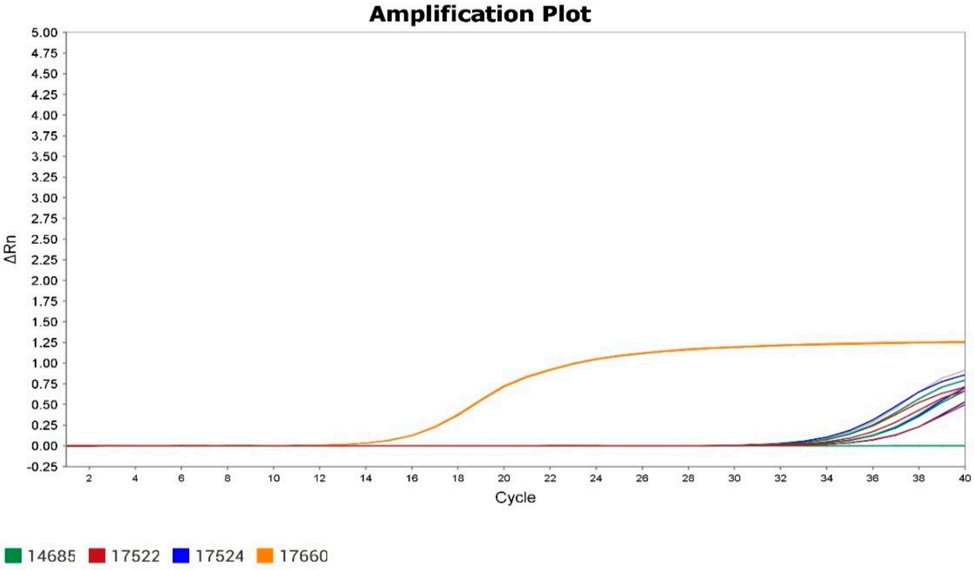

(b)

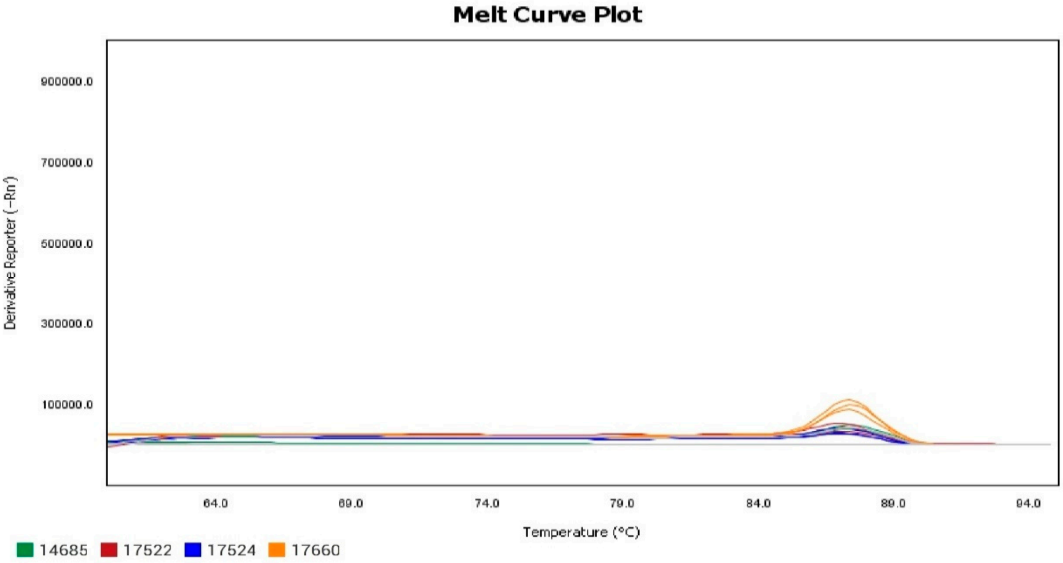

Figure S5. Test results of *vdcC* primer using TAKARA TB GREEN reagent. (a) Amplification plot. (b) Melting curves.

The Ct value of *A. acidoterrestris* BCRC 17660 was 15.561, the Ct value of *A. cycloheptanicus* BCRC 17522 was 35.266, the Ct value of *A. hesperidum* BCRC 17524 was 35.266, and the Ct value of *A. acidocaldarius* BCRC 14685 was 34.956. The Tm value of *A. acidoterrestris* BCRC 17660 was 87.304, the Tm value of *A. cycloheptanicus* BCRC 17522 was 87.012, the Tm value of *A. hesperidum* BCRC 17524 was 87.060, and the Tm value of *A. acidocaldarius* BCRC 14685 was 87.447.

(a)

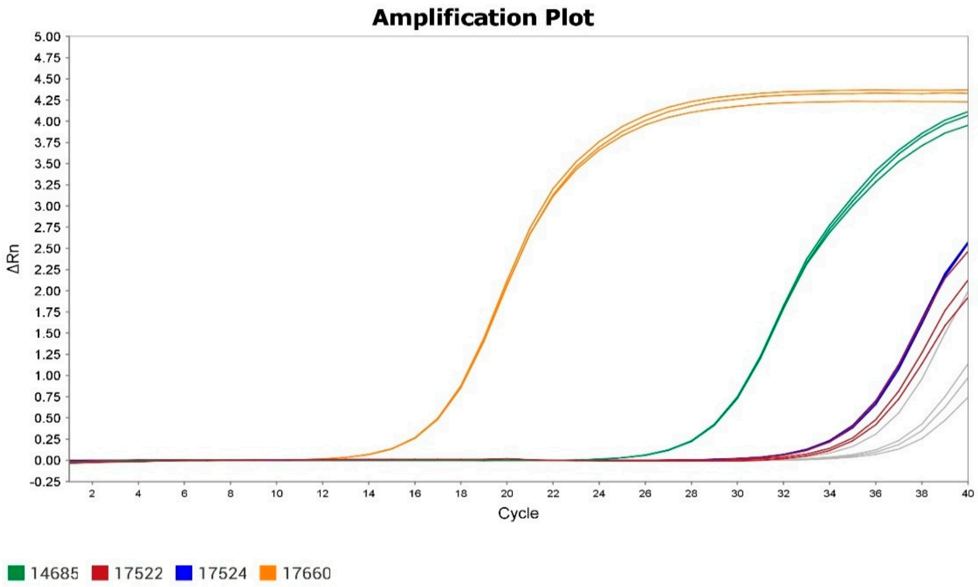

(b)

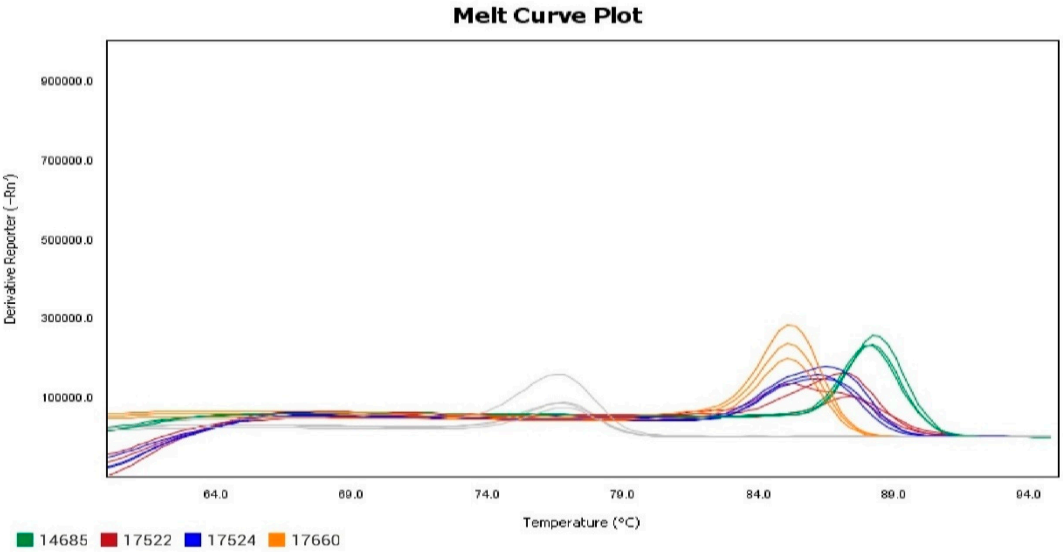

Figure S6. Test results of *vdcC* primer using KAPA SYBR GREEN reagent.

(a) Amplification plot. (b) Melting curves.

The Ct value of *A. acidoterrestris* BCRC 17660 was 16.202, the Ct value of *A. cycloheptanicus* BCRC 17522 was 35.040, the Ct value of *A. hesperidum* BCRC 17524 was 34.502, and the Ct value of *A. acidocaldarius* BCRC 14685 was 28.446. The Tm value of *A. acidoterrestris* BCRC 17660 was 85.140, the Tm value of *A. cycloheptanicus* BCRC 17522 was 86.516, the Tm value of *A. hesperidum* BCRC 17524 was 86.326, and the Tm value of *A. acidocaldarius* BCRC 14685 was 88.177.
